# Abnormal uterine bleeding

**DOI:** 10.1016/j.bpobgyn.2015.11.012

**Published:** 2016-07

**Authors:** Lucy Whitaker, Hilary O.D. Critchley

**Affiliations:** MRC Centre for Reproductive Health, University of Edinburgh, Edinburgh EH16 4TJ, UK

**Keywords:** abnormal uterine bleeding (AUB), fibroids, FIGO PALM-COEIN classification of AUB

## Abstract

Abnormal uterine bleeding (AUB) is a common and debilitating condition with high direct and indirect costs. AUB frequently co-exists with fibroids, but the relationship between the two remains incompletely understood and in many women the identification of fibroids may be incidental to a menstrual bleeding complaint. A structured approach for establishing the cause using the Fédération International de Gynécologie et d'Obstétrique (FIGO) PALM-COEIN (**P**olyp, **A**denomyosis, **L**eiomyoma, **M**alignancy (and hyperplasia), **C**oagulopathy, **O**vulatory disorders, **E**ndometrial, **I**atrogenic and **N**ot otherwise classified) classification system will facilitate accurate diagnosis and inform treatment options. Office hysteroscopy and increasing sophisticated imaging will assist provision of robust evidence for the underlying cause. Increased availability of medical options has expanded the choice for women and many will no longer need to recourse to potentially complicated surgery. Treatment must remain individualised and encompass the impact of pressure symptoms, desire for retention of fertility and contraceptive needs, as well as address the management of AUB in order to achieve improved quality of life.

## Highlights

• The FIGO ‘PALM COEIN’ classification of AUB is considered in this review
• Mechanisms by which fibroids contribute to AUB are elucidated.
• A structured approach to management of the patient with fibroids and AUB is proposed.

## Background

Abnormal uterine bleeding (AUB) is a significant clinical entity. AUB and its sub group, heavy menstrual bleeding (HMB), are common conditions affecting 14–25% of women of reproductive age [1], [2] and may have a significant impact on their physical, social, emotional and material quality of life [3]. In the UK, over 800,000 women seek help for AUB annually [3]. Along with the direct impact on the woman and her family, there are significant costs to both economy and health service. A US study reported financial losses of >$2000 per patient per annum due to work absence and home management costs [4]. AUB is the fourth most common reason for referral to UK gynaecological services [5]. A recent national audit in England and Wales (RCOG HMB audit) reported that at 1-year post referral, only a third of women (including those managed with surgery) were ‘satisfied’ (or better) at the prospect of current menstrual symptoms continuing, as currently experienced, for the next 5 years [6]. While there may be relief from HMB during pregnancy and lactation, and an end to the problem at menopause, women affected will tend to suffer the adverse impacts of AUB over what should be the prime years of their lives.

Fibroids (leiomyoma) represent the most common tumour of women; by the age of 50, almost 70% of white women and >80% of black women will have developed at least one fibroid [7]. Fibroids are associated with subfertility, miscarriage, preterm labour and obstruction of labour. In addition, they may cause discomfort and pressure symptoms, typically urinary. In rare circumstances, at larger sizes, they may cause compression of the renal tract and pelvic vasculature leading to impaired renal function and venous thromboembolism, respectively. Conversely, many women with fibroids will be entirely asymptomatic [8]. However, many women most commonly present to gynaecological services with AUB and associated iron-deficiency anaemia. For women with uterine fibroids, everyday life is often disrupted and fibroids remain a leading indication for hysterectomy [9], [10]. Conservative estimates of annual direct treatment costs and indirect costs from lost work hours as a result of fibroids are $4.1–9.4 billion and $1.55–17.2 billion, respectively [11]. The mechanisms, however, linking AUB and fibroids remain incompletely understood.

As women increasingly defer pregnancy, fertility preservation is critical and newer medical options offer genuine effective relief for both AUB and other symptoms associated with fibroids. This review addresses the causes of AUB and approach to assessment and general principles of management of the pre-menopausal woman with fibroids.

## Definitions

AUB was redefined by Fédération International de Gynécologie et d'Obstétrique (FIGO) in 2009 by the FIGO Menstrual Disorders Group (FMDG) [12], ∗[13]. This was in order to standardise definitions, nomenclature and the underlying categories of aetiology. It was hoped that this would facilitate ease of investigation and comparison of similar patient populations and thereby aid research and improve evidence-based care; this would also be a practical tool for assessing contributing aetiologies.

Chronic AUB was defined as ‘bleeding from the uterine corpus that is abnormal in volume, regularity and/or timing that has been present for the majority of the last 6 months’ [13]. Values outwith the accepted 5–95th percentiles indicated abnormality (Table 1).

With regard to volume, however, both the Royal College of Obstetricians and Gynaecologists (RCOG) and American College of Obstetricians and Gynecologists (ACOG) prefer the patient-centred definition of HMB, ‘excessive menstrual blood loss which interferes with a woman's physical, social, emotional and/or material quality of life’ [3], as an indication for investigation and treatment options. As such, objective measurements of volume are usually the preserve of research studies and surrogates such a pictorial blood-loss assessment chart (PBAC) scores are not recommended in routine clinical practice.

## FIGO classification of cause: ‘PALM-COEIN’

**Fig. 1:**
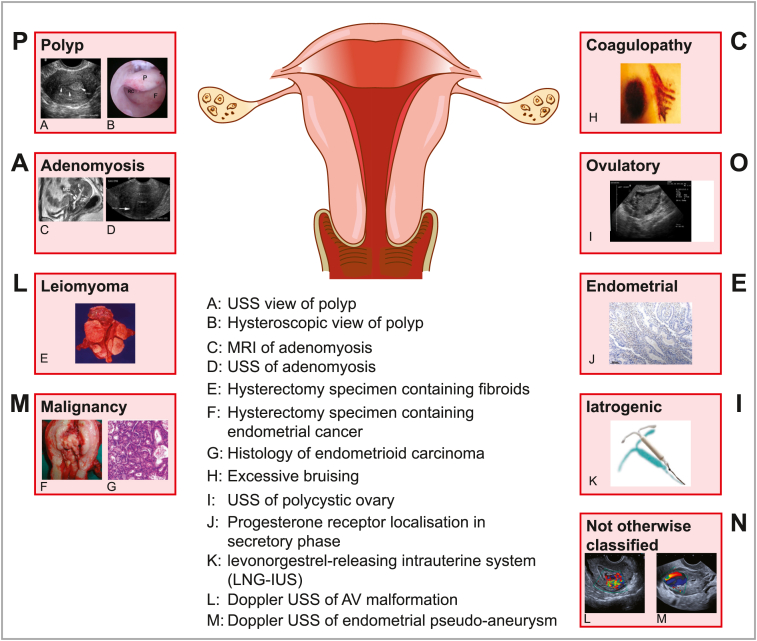
FIGO classification of causes of AUB; ‘PALM COEIN’.

Once bleeding is defined as being abnormal, the acronym PALM-COEIN is now being increasingly used for categorising causes: **P**olyp, **A**denomyosis, **L**eiomyoma, **M**alignancy (and hyperplasia), **C**oagulopathy, **O**vulatory disorders, **E**ndometrial, **I**atrogenic and **N**ot otherwise classified [13]. The ‘PALM’ are assessed visually (imaging and histopathology) and the ‘COEIN’ are non-structural (Fig. 1).

**Fig. 2:**
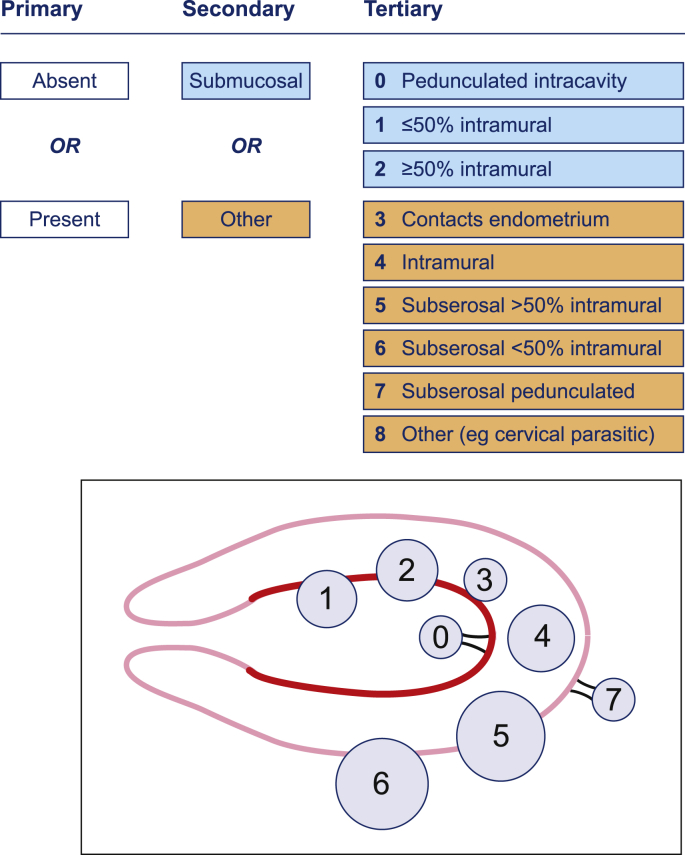
Tertiary classification of AUB-L (adapted from Munro et al. [13]).

Depending on the site, leiomyoma (fibroids) are further subdivided into submucosal (SM) and other (O) and then into nine tertiary categories adapted from the Wamsteker classification [14] (Fig. 2). These have been adopted by the European Society for Human Reproduction and Embryology (ESHRE) and used by the European Society for Gynaecological Endoscopy (ESGE).

## Contribution of fibroids (leiomyoma) to AUB

The relationship between AUB and fibroids remains incompletely understood. The obvious paradox is that many women have fibroids but also have entirely normal bleeding patterns. Fibroids are also highly prevalent in women presenting with AUB.

Previous postulated theories include an increased endometrial surface area and the presence of fragile and engorged vasculature in the perimyoma environment [15]. The increase in vascular flow observed along with these enlarged vessels can overcome platelet action [16]. There is increasing knowledge regarding the complex cellular and molecular changes found in association with fibroids, with impact on angiogenesis, alteration in vasoactive substrates and growth factors as well as alteration in coagulation [16]. The effect of fibroids on endometrial function is now thought to represent a field change within the uterine cavity rather than limited to regions overlying the myoma(s). Some of these changes may have an impact on endometrial receptivity and implantation as well as AUB [17], ∗[18].

Matrix metalloproteinase (MMP) 2 and 11 levels are increased in fibroids (with MMP 1 and 3 unchanged) [19], [20], but the impact on endometrial bleeding is unclear. Expression of vascular endothelial growth factor (VEGF), basic fibroblast growth factor (bFGF), heparin-binding epidermal growth factor, platelet-derived growth factor (PDGF), parathyroid hormone-related protein (PTHrP) and prolactin is altered in women with fibroids [16]. VEGF, bFGF, PDGF and PTHrP all have potential angiogenic effects but their specific role within the endometrium in women with fibroids has yet to be determined [17].

There is alteration of plasminogen modulators and this may impact on endometrial haemostasis and repair [16]. Transforming growth factor beta (TGF-β) is produced in excess in the endometrium in women with fibroids and is associated with reduced levels of plasminogen activator inhibitor-1 (PAI-1), thrombomodulin and antithrombin III, both in vivo and in endometrial stromal cells treated in vitro with TGF-β [18]. This may represent a putative mechanism for some cases of AUB observed in the context of fibroids and may in the future offer a potential therapeutic target.

In women with fibroids, alterations in the blood plasma levels of circulating interleukin (IL)-13, IL-17 and IL-10 have been reported [21]. Whether these variations affect immune function and inflammation implicated in endometrial breakdown and repair remains unknown.

With regard to the location of fibroids, it was previously thought that those women with SM fibroids, particularly with those distorting the cavity, were more likely to present with HMB [15]. There is current debate that women with significant cavity distortion represent additional therapeutic challenges.

## Other causes of AUB

The PALM-COEIN classification system accepts that women may have more than one underlying aetiology and also that often in the case of structural abnormalities, many women may in fact be asymptomatic.

### Polyps (AUB-P)

Endometrial polyps are epithelial proliferations arising from the endometrial stroma and glands. The majority are asymptomatic. The contribution of polyps to AUB varies widely ranging from 3.7% to 65% [22], [23], but it is widely accepted [24]. The incidence of polyps as with fibroids increases with age and both pathologies may frequently co-exist, or suspected polyps visualised on transvaginal ultrasound scanning (TV-USS) may be mistaken for SM fibroids and vice-versa [25].

### Adenomyosis (AUB-A)

The relationship between adenomyosis and AUB remains unclear [26], particularly with regard to wide variations in histopathological diagnosis reflecting variations in criteria used and also improved radiological diagnosis. Typically, adenomyosis is associated with increasing age and may co-exist with fibroids. Furthermore, adenomyosis may be both focal and diffuse and it may be harder to establish diagnosis if fibroids are also present [27].

### Malignancy (AUB-M)

Endometrial cancer is the most common gynaecological malignancy in the western world. Historically, endometrial cancer has rarely occurred in premenopausal women; however, with increasing obesity and rising prevalence of the metabolic syndrome, the endocrine-driven subset of endometrial malignancy has markedly increased in frequency. Between 1992–1994 and 2009–2011, the European age-standardised rates of uterine cancer in the UK have increased by 48% [28]. With the reclassification by the WHO from hyperplasia to endometrial intraepithelial neoplasia (EIN), the current prevalence of premalignant disease is unknown. The evaluation of the endometrium may be affected by distortion of the uterine cavity by fibroids, and as such, the co-existing pathology may delay diagnosis.

The diagnosis of cervical cancer should be considered, particularly with persistent intermenstrual bleeding, and rarely ovarian cancer may present with AUB.

Uterine sarcoma have been reported as rare (3–7/100,000 in the USA) [29] but maybe a cause of AUB-M. A recent meta-analysis reported that leiomyosarcoma are unexpectedly diagnosed following surgery for anticipated ‘benign’ myomas in 2.94 per 1000 women (one in 340 women) [30], [31]. Race is the only commonality between leiomyosarcoma and leiomyoma with black women having an approximately twofold increased risk [29]. The risk of development of leiomyosarcoma is reported to increase with age with <1 case per 500 among women aged under 30 years to one in 98 among women in the age range 75–79 years [30], [31]. Other risk factors for uterine leiomyosarcoma include the long-term use of tamoxifen [32], previous pelvic radiation therapy [33] and rare inherited disorders such as hereditary leiomyomatosis and renal cell carcinoma (HLRCC) [34].

Interestingly, the previously held view was that a rapidly enlarging uterus would raise the suspicion for malignancy. This is now no longer held to be true as benign fibroids can grow rapidly and sarcomas grow slowly [35], [36]. However, more objective investigations are still lacking. Both ultrasound scanning (USS) and magnetic resonance imaging (MRI) do not as yet have robust criteria to accurately predict differentiation between leiomyoma and leiomyosarcoma [37]. The lack of a robust pre-surgical predictor/biomarker has recently altered surgical practice because morcellation of an unsuspected leiomyosarcoma increases dissemination [38].

If malignancy or premalignancy is found along with AUB classification, the pathology should be described and staged utilising the appropriate WHO/FIGO systems [39].

### Coagulopathy (AUB-C)

Coagulopathies are reported to affect 13% [40] of the women presenting with HMB. The majority of these women suffer from Von Willebrand disease [40]. Systemic disorders of haemostasis may be identified in 90% of women using a structured history [41], ∗[42] (Table 2).

If 1, 2 or 3 (see Table 2) is ascertained, it indicates positive screen, and further referral for appropriate investigation should be considered.

Anticoagulant and antiplatelet therapy hitherto has been considered as a part of ‘AUB-C’ (rather than AUB-I). Compression caused by a large fibroid uterus may lead to venous thromboembolism (VTE). Bleeding previously deemed as AUB-L may be exacerbated by subsequent anticoagulation and presents additional management challenges.

### Ovulatory (AUB-O)

Anovulatory cycles may contribute to AUB by unopposed oestrogen effects on the endometrium causing marked proliferation and thickening resulting in HMB along with an altered frequency of menstruation. This is observed at the extremes of reproductive age; however, impact on the HPO axis along with endocrinopathies is also present. The latter include polycystic ovarian syndrome (PCOS), hyperprolactinaemia, hypothyroidism as well as factors such as obesity, anorexia, weight loss, mental stress and extreme exercise. Typically, women in this group have menstrual cycles that fall out with 38 days or have a variation of >21 days. Drugs that affect dopamine levels, with their attendant effects on the HPO axis, also currently fall under this category rather than AUB-I. In women with fibroids, the co-existing ovulatory dysfunction may exacerbate menstrual loss.

The FIGO AUB classification system is a dynamic system with feedback and contemporary debate informing future revisions [13]. The position of drug therapies affecting AUB is currently under review with regard to whether anticoagulant/antiplatelet therapies and drugs affecting the HPO axis may be better placed in ‘AUB-I’.

### Endometrial (AUB-E)

AUB that occurs in the context of a structurally normal uterus with regular menstrual cycles without evidence of coagulopathy is likely to have an underlying endometrial cause. Endometrial function in the context of menstruation and its disorders is still not fully understood and remains an area of active scientific enquiry, particularly the complexities of the sequence of events triggered by progesterone withdrawal (due to demise of the corpus luteum in the absence of pregnancy). Hypoxia, inflammation, haemostasis and angiogenesis all play crucial roles in the shedding and subsequent scarless repair of the functional upper layer of the endometrium. Perturbation of local glucocorticoid metabolism, aberrant prostaglandin synthesis and excessive plasminogen (resulting in premature clot lysis) have all been implicated in AUB [43].

AUB-E may be implicated in many women with AUB, but a lack of clinically available specific tests or biomarkers means that practical testing for such disorders is not yet feasible. As such, diagnosis depends on careful history taking and exclusion of other contributors. The high prevalence of potential endometrial dysfunction means that it is highly likely that those with AUB-L will often have an element of AUB-E contributing to increased/aberrant menstrual blood loss with its attendant implication for therapy.

### Iatrogenic (AUB-I)

Iatrogenic causes of AUB include exogenous therapy than may lead to unscheduled endometrial bleeding. This is typically associated with continuous oestrogen or progestin therapy (systemic or intrauterine delivery routes) or those interventions that act on ovarian steroid release such as gonadotropin-releasing hormone (GnRH) agonists and aromatase inhibitors. Selective oestrogen receptor modulators (SERMs) and more rarely selective progesterone receptor modulators (SPRMs) may cause AUB through direct action on the endometrium.

The use of an intrauterine device (IUD) may cause a low-grade endometritis which may also contribute to AUB.

### Not otherwise classified (AUB-N)

It is inevitable that there will be pathologies that are either rare or poorly defined that do not easily fit within the categories described earlier. Examples include arteriovenous malformations, endometrial pseudoaneurysms, myometrial hypertrophy and chronic endometritis (not precipitated by an IUD). All of these can co-exist with AUB-L.

The planned regular review of the FIGO PALM-COEIN classification system every 3–5 years through FIGO [13] will allow reassessment, in particular, of this category. Further areas considered for future sub-classification include AUB-P and AUB-A.

## Assessment of the patient presenting with AUB and fibroids

As described earlier, all of the other causes of AUB may co-exist with fibroids. As such, it is crucial when a patient with known or suspected fibroids presents with AUB, she is appropriately assessed for the presence of other aetiologies. Misdiagnosis will have an impact on treatment options and efficacy, and in the event of undiagnosed coagulopathy, render surgical intervention disproportionately hazardous.

**Fig. 3:**
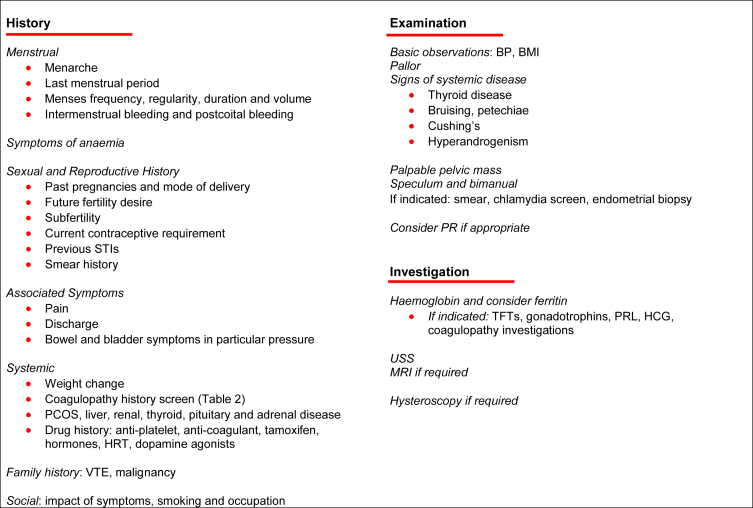
Structured approach for assessing the patient presenting with AUB.

As part of structured history, factors such as co-morbidities, polypharmacy, body mass index (BMI), previous surgery and most crucially fertility desire and impact of pressure symptoms must be assessed as these significantly affect treatment approach. A structured approach is shown in Fig. 3.

An accurate menstrual history and associated symptoms will identify a likely AUB-O cause. As described earlier, a structured screen for coagulopathies will identify 90% of those women with disorders of systemic haemostasis (Table 2). History will also identify contributors to AUB-I.

Combined history and examination will suggest possible AUB-P/-A/-L and should be confirmed with subsequent imaging. TV-USS remains the most acceptable and validated first-line investigation. The increasing use of saline infusion ultrasonography (SIS) and selected hysteroscopy will improve sensitivity and specificity for diagnosis of polyps and SM fibroids ∗[37], ∗[44]. The optimal mode of imaging for suspected adenomyosis has yet to be established [45]. Furthermore, women with fibroids may have them confused for focal adenomyosis and vice-versa using conventional imaging [27]. The increased use of one-stop clinics with access to outpatient hysteroscopy improves patient satisfaction and facilitates timely investigation and management [46].

MRI plays a role in selected patients with AUB and fibroids, also in the assessment of suitability for uterine artery embolisation (UAE). As previously discussed, it is relatively poor at providing reassurance of the absence of sarcomatous change.

### Endometrial sampling

In the UK, NICE recommend endometrial sampling in women with persistent inter-menstrual bleeding or aged ≥45 years with treatment failure [3]. This has been highlighted in the RCOG guidelines with an exception of reducing the age of sampling in the context of treatment failure to 40 [47]. With the marked increase in endometrial cancer, the authors would encourage all gynaecologists to continue to excise their clinical judgement for those women aged <40 years with HMB who have risk factors for premalignant change such as obesity and PCOS. Endometrial sampling may be more challenging if fibroids distort the cavity, and access to concurrent outpatient hysteroscopy can facilitate timely exclusion of endometrial pathology.

## Approach to management

Management of AUB-L should address fertility desire, impact of pressure symptoms, co-morbidities, and any other AUB contributors. Treatment should be individualised. No one-size-fits-all approaches are available with regard to initial and subsequent treatment options, and there is a relative paucity of large robust clinical trials providing head-to-head data rather to placebo.

In those with other underlying AUB causes co-existing with fibroids, targeted treatment of these may ameliorate bleeding, and in the absence of pressure symptoms or sub-mucosal myoma-related infertility, all the treatments may be required. Specific treatments for other causes are shown in Table 3.

**Fig. 4:**
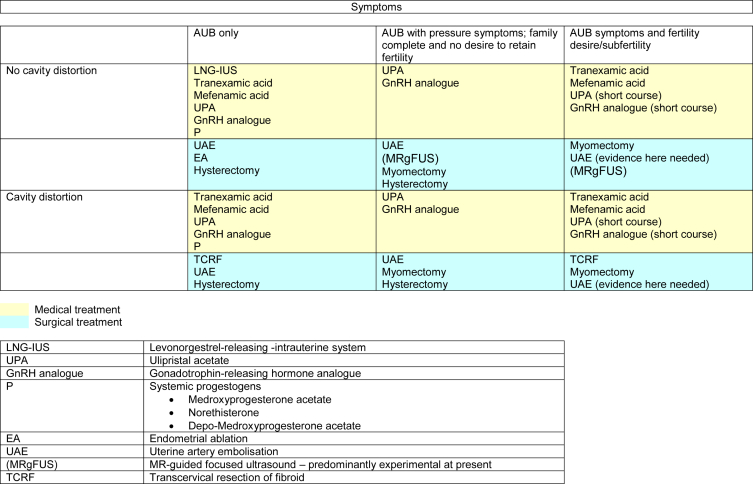
Symptom-based approach for management of AUB in the context of fibroids.

Otherwise, treatment should be tailored depending on the impact of related symptoms, fertility requirements and cavity distortion (Fig. 4). It should be remembered that a conservative approach (incorporating oral iron replacement if indicated) may be an entirely acceptable treatment approach, particularly in the peri-menopausal phase with amenorrhea and regression of fibroid size imminent.

In AUB, in the absence of pressure symptoms, medical treatment may be more appropriate, particularly when fertility preservation is required. Tranexamic acid and NSAIDs (e.g. mefenamic acid) remain the only fully non-contraceptive medical options [3]. Whilst the risk of expulsion of a levonorgestrel-releasing intrauterine system (LNG–IUS) is without doubt higher in the context of fibroids, there is still evidence for efficacy [48] although cavity distortion may preclude the use of LNG–IUS.

The current Cochrane review for the SPRMs is limited to mifepristone [49] and a future review incorporating other members of the SPRM class is underway. GnRH analogues are effective in reducing both size of fibroids and amelioration of bleeding, but their side effects and impact on bone density limit their longer-term utility, and rebound of symptoms is rapid on cessation [50]. GnRH agonists often are beneficial as a short-term treatment prior to IVF or surgery, but given the findings in the PEARL II study, there is good evidence that the SPRM ulipristal acetate (UPA) is better tolerated in those women pre-surgery without loss of efficacy [51]. There is no robust evidence for alternative therapies such as acupuncture or herbal remedies for the treatment of fibroids [52], [53].

With regard to interventional radiological (uterine artery embolisation, UAE) and surgical options, the anticipated outputs of the FEMME study [54] will hopefully provide robust evidence for impact on symptoms and other qualitative measures between myomectomy and UAE. MR-guided focussed ultrasound (MRgFUS) is not a widely available technique. Its role in the management of symptomatic fibroids remains to be established. Hysterectomy is a definitive treatment, and in the context of management options for HMB, it remains as a therapeutic option with the highest patient satisfaction and cost-effectiveness for >5 years [55]. Hysterectomy, however, is often a challenging surgery in women with high potential blood losses and risk of ureteric injury due to anatomical distortion in the pelvis. With increasing obesity, the complexity of surgery is compounded. Whilst alternative treatment strategies are under development, a cohort of women whose fertility plans are complete and for whom definitive surgery, that is, hysterectomy, becomes the most appropriate management will remain.

## Conclusions

AUB is a common and debilitating condition with high direct and indirect costs. Symptoms of AUB frequently co-exist with fibroids, but the relationship between AUB and fibroids remains incompletely understood. In many women, fibroids may be an incidental innocent bystander in the underlying aetiology of a menstrual bleeding complaint. A structured approach to establishing the cause using the FIGO PALM-COEIN classification system will facilitate accurate diagnosis and inform treatment options. The classification system, however, still lacks effective biomarkers for ‘AUB-E’. Office hysteroscopy and the increasingly sophisticated imaging will assist provision of robust evidence for the underlying cause. The increased availability of medical options has expanded the choice for women. Many will no longer need to recourse to potentially complicated surgery. Treatment must remain individualised and encompass the impact of pressure symptoms, desire for retention of fertility and contraceptive needs, as well as address the management of their AUB in order to achieve improved quality of life.

**Practice points**

• A structured approach for using the PALM-COEIN framework should be developed to ensure that important contributors apart from fibroids are not missed.
• In particular, coagulopathies should be verbally screened for in all patients presenting with AUB, given their high prevalence in this population and implications for management.
• In view of the rapid increase in endometrial cancer, clinical judgment regarding endometrial sampling should be considered in younger women with AUB with risk factors for EIN and malignancy.
• Treatment should be individualised encompassing impact of pressure symptoms, desire for retention of fertility, contraceptive needs and impact of symptoms on quality of life.

**Research agenda**

• Imaging for fibroids, in particular, modality for diagnosis of adenomyosis and for discriminating between fibroids and leiomyosarcoma.
• Increased evidence base for long-term medical treatments for management of AUB in the context of fibroids, with particular emphasis on quality of life.
• Improved evidence for interventional treatments for AUB in the context of fibroids.

## Conflict of interest statement

LW has no conflict of interest. HODC has clinical research support for laboratory consumables and staff from Bayer Pharma AG and provides consultancy advice (but with no personal remuneration) for Bayer Pharma AG, PregLem SA, Gedeon Richter, Vifor Pharma UK Ltd, AbbVie Inc.

## Figures and Tables

**Table 1:** Suggested Normal limits for menstrual parameters. Adapted from Fraser et al. [12].

Clinical Parameter	Descriptive term	Normal limits (5–95th percentiles)
Frequency of menses (days)	Frequent Normal Infrequent	<24 24–38 >38
Regularity of menses, cycle to cycle (Variation in days over 12 months)	Absent Regular Irregular	No bleeding Variation ± 2–20 days Variation >20 days
Duration of flow (days)	Prolonged Normal Shortened	>8.0 4.5–8.0 <4.5
Volume of monthly blood loss (mL)	Heavy Normal Light	>80 5–80 <5

**Table 2:** Structured history for coagulopathy screen. Adapted from Koudies et al. [42].

Criteria
1. Heavy bleeding since the menarche
2. One of the following: • Postpartum haemorrhage • Surgical-related bleeding • Bleeding associated with dental work
3. Two or more of the following: • Bruising 1–2 times/month • Epistaxis 1–2 times per/month • Frequent gum bleeding • Family history of bleeding problems

**Table 3:** Specific treatment options for individual PALM-COEIN causes of AUB.

AUB Sub-classification	Specific treatment
Polyp	Resection
Adenomyosis	Surgery: hysterectomy; adenomyomectomy (not frequently performed)
Malignancy	Surgery +/− adjuvant treatment High-dose progestogens (if surgery not possible) Palliation (including radiotherapy)
Coagulopathy	Tranexamic acid DDVAP
Ovulation	Lifestyle modification Cabergoline (if hyperprolactinaemia) Levothyroxine (if hypothyroid)
Endometrial	Specific therapies await further delineation of underlying mechanisms
Iatrogenic	Refer to FSRH CEU guidance on problematic bleeding with hormonal contraception [56]
Not otherwise classified	Antibiotics for endometritis Embolisation of AV malformation
